# Remedy for Whooping Cough

**Published:** 1873-12

**Authors:** 


We have received from our friend Dr. J. F. Hammond, of this city, the following formula for Whooping Cough, which he regards as well nigh *specific* in that disease. It is as follows:

℞.—Ammon. Bromid., . . . . ʒj.

Tinct. Lobeliæ, . . . . . Gtt. xx.

Tinct. Stramonii . . . . Gtt. iv.

Acid. Hydrocyan. Dil., . . . Gtt. vj.

Syr. Pruni Virg., . . . . ℥j.

Elix. Cort. Aurant, . . . . ʒij.

Aquæ, . . . . . . ʒvj

M. Ft. Sol.

Sig.—For child 8 to 12 months old, 10 to 20 drops every 3 or 4 hours.

